# *In situ* Orchid Seedling-Trap Experiment Shows Few Keystone and Many Randomly Associated Mycorrhizal Fungal Species During Early Plant Colonization

**DOI:** 10.3389/fpls.2018.01664

**Published:** 2018-11-16

**Authors:** Stefania Cevallos, Stéphane Declerck, Juan Pablo Suárez

**Affiliations:** ^1^Laboratory of Mycology, Earth and Life Institute, Université catholique de Louvain, Louvain-la-Neuve, Belgium; ^2^Departamento de Ciencias Biológicas, Universidad Técnica Particular de Loja, Loja, Ecuador

**Keywords:** biotic and abiotic factors, keystone mycorrhizal, mycorrhizal fungi, seedling-trap experiment, temporal variation

## Abstract

Orchids are known for their vast diversity and dependency on mycorrhizal fungi. Under *in situ* conditions, the biotic and abiotic factors determining the composition and distribution of orchid mycorrhizal fungi (OMF) communities remain largely unexplored. Therefore *in situ* experiments are needed to better understand the interactions between orchids and fungi. A seedling-trap experiment was conducted in the Reserva Biológica San Francisco, a well-known biodiversity hotspot located in the Andes of southern Ecuador. The objective was to investigate the effect of orchid species, site, elevation or temporal variation on the assembly and structure of OMF associated with *Cyrtochilum retusum* and *Epidendrum macrum*. The OMF community composition was determined using the Illumina MiSeq sequencing of the internal transcribed spacer 2 (ITS2) region. The results exhibited 83 OMF operational taxonomic units belonging to Tulasnellaceae, Ceratobasidiaceae, Serendipitaceae and Atractiellales. It was observed that the composition of the OMF communities was different among orchid species and temporal variation but was not different among sites. The results further support that orchids have a core of keystone OMF that are ubiquitously distributed and stable across temporal change, whereas the majority of these fungi are randomly associated with the plants.

## Introduction

In nature, orchids rely on particular interactions with their pollinators and root fungal associates (i.e., the mycorrhizal fungi) for completing their life cycle (Selosse, 2014). Orchid mycorrhizal fungi (OMF) influence plant development at different life stages (Cameron et al., 2006). For instance, the tiny seeds of orchids lack carbohydrate reserves, making them dependent on mycorrhizal fungi for germination and subsequent development into protocorms (Smith and Read, 2008).

It is widely accepted that local abundance and population dynamics of orchids are largely dependent on mycorrhizal fungi (McCormick and Jacquemyn, 2014). However, even if an increasing number of OMF have been identified (Kartzinel et al., 2013; Kohout et al., 2013), their community structures and the factors affecting their spatial distribution in natural environments remain poorly explored (McCormick and Jacquemyn, 2014).

Across the last three decades, our knowledge on the diversity and community composition of OMF has increased markedly with the development of powerful sequencing technologies (Merckx, 2013). With Sanger sequencing, fungi within the Tulasnellaceae (Suárez et al., 2006, 2016; Riofrío et al., 2013; Herrera et al., 2018), Serendipitaceae (Suárez et al., 2008), Ceratobasidiaceae (Otero et al., 2004) and Atractiellales (Kottke et al., 2010) were reported as dominant associates of tropical orchids. The development of next-generation sequencing (NGS) technologies (i.e., 454 and Illumina) has further helped to improve the characterization of fungal communities and assess their ecological dynamics (Cardenas and Tiedje, 2008). For instance, Cevallos et al. (2017) and Herrera et al. (in press) demonstrated that OMF communities were site-adjusted, consisting of a core of generalists and ubiquitous orchid mycorrhizal fungi-operational taxonomic units (OMF-OTUs), considered as keystone species (stable component) plus OTUs identified at specific-site orchid populations (dynamic component). This indicates that the structure of the dynamic component of the OMF communities is determined by local environmental conditions and host’s evolutionary history and that keystone species occur irrespective of the orchid species or site. Likewise, in temperate regions, significant differences in OMF communities were reported across orchid species (Esposito et al., 2016) and through temporal variation (Oja et al., 2015) with a core of generalist and overlapped OMF communities.

A number of biotic (i.e., orchid species) and abiotic (i.e., site, temporal variation) factors have been reported to impact the structure of OMF communities (Jacquemyn et al., 2012; Ercole et al., 2015; Esposito et al., 2016; Cevallos et al., 2017). However, it is often difficult to generalize the ecological premises to all orchid species or geographic regions, mainly because particular combinations of environmental factors may markedly influence the local OMF community structure (Peay et al., 2010). Most data on the orchid-fungi assemblages come from *in vitro* experiments (Těšitelová et al., 2012; Fracchia et al., 2016) and orchid roots sampled from their natural habitats at a specific spatial or temporal scale (Oja et al., 2015; Mujica et al., 2016). In contrast, field experiments to study the orchid-fungal symbiosis are less numerous, probably because of the morphological and biological characteristics of orchids (i.e., minute seeds, nutritional requirements) that make it difficult to conduct *in situ* investigations (Bayman et al., 2002). Compared to *in vitro* studies, it seems obvious that field experiments could provide more realistic insights into the ecology and evolutionary patterns of orchid-fungal interactions (Bidartondo and Read, 2008; Phillips et al., 2011; Waterman et al., 2011; McCormick et al., 2012; Těšitelová et al., 2012).

The evolutionary history of orchids is considered one of the key factors that shape the structure of the OMF community. With more closely related orchid species, more similar OMF communities are predicted (Těšitelová et al., 2015; Cevallos et al., 2017). In co-existence, related orchid species tend to be associated with similar mycorrhizal fungi (Suárez et al., 2016), although the entire OMF community structure could be different (McCormick and Jacquemyn, 2014). In addition, the site (where each orchid population is present) has also been considered as a driver of the composition of OMF communities. Depending of the spatial scales, the combination of fungal dispersal limitations, biogeographic history and adaptive evolution create a unique fungal assemblage (Peay et al., 2010). Elevation also affects the OMF communities. Although little information is available, it has been reported that OMF communities change with increasing elevation and that the peak of OMF richness occurs at the mid-elevation in montane forest (Jacquemyn et al., 2005; Geml, 2017). On a temporal scale, OMF communities change because of the orchid nutritional demands across the life cycle (Smith and Read, 2008; Ercole et al., 2015). Temporal dynamics of the orchid-fungi symbiosis have been evaluated across seasonal and environmental conditions as well as orchid developmental stages (i.e., fruiting, flowering) (Rasmussen and Whigham, 2002; Shefferson et al., 2005; Ercole et al., 2015). However, it remains unclear whether the temporal shifts of OMF partners are a consequence of the succession or represent opportunistic associations due to the extrinsic conditions (Otero et al., 2007; Oja et al., 2015). The evidence available thus far shows that it is unlikely that a single factor is responsible for the structure and composition of OMF communities (McCormick and Jacquemyn, 2014).

In natural orchid populations, the evaluation of the influence of multiple biotic or abiotic factors (i.e., orchid species, temporal variation) on OMF communities is not always possible, in consideration of the particular distribution and life cycle that each orchid species has. When orchids are more widely distributed in specific areas, the implementation of field experiments provides opportunities to study the effect of multiple environmental factors on the communities of OMF (Phillips et al., 2011). Here, a field experiment was conducted using *in vitro* seedlings of *Cyrtochilum retusum* (Lindl.) Kraenzl. and *Epidendrum macrum* Dressler, two epiphytic orchids present in southern Ecuador. The orchid species were established along an elevational gradient in two sites of the Podocarpus National Park (southern Ecuador). The OMF communities of the *in vitro* seedlings were determined by Illumina MiSeq amplicon sequencing analysis. This provided a unique opportunity to elucidate whether co-existing orchid species, site, elevational level and temporal variation have an effect on the assembly and structure of mycorrhizal communities *in situ*.

## Materials and Methods

### Study Site

The study was conducted in the Reserva Biológica San Francisco (RBSF), a well-known biodiversity hotspot, located in the eastern Andes of southern Ecuador (Myers et al., 2000) and classified as tropical montane forest (Beck et al., 2008). The vegetation is characterized by an exceptional richness of plant families such as Orchidaceae (337 spp.) and Lauraceae (40 spp.) (Homeier et al., 2008). The mean annual rainfall and temperature are 2000 mm and 15.5°C, respectively. The rainy season extends from April to August and the dry season from September to March (Table 8.7 in Beck et al., 2008). The seedling-trap experiment was set up in the lower area of the RBSF, between 1850 and 2150 m.a.s.l. in two sites known as T2 a ridge forest (3°58.415^′^S, 79°04.516^′^W) and Q5 a ravine forest (3°58.399^′^S, 79°04.243^′^W) (Bussmann, 2003). The distance between T2 and Q5 is about 1 km. The transects were selected because of the data available on the environment (i.e., climate, flora) (Beck et al., 2008).

The seedling-trap experiment was established from May 2014 to April 2015 using *in vitro-*produced seedlings of *Cyrtochilum retusum* (Lindl.) Kraenzl. (identified as C in the treatments coding) and *Epidendrum macrum* Dressler (identified as E in the treatments coding) (tribes Cymbidieae and Epidendreae, respectively) obtained from a private seller (Ecuagenera, Azuay-Ecuador). Individuals of *C. retusum* have been observed in Loja and Zamora-Chinchipe provinces between 1700 and 3150 m.a.s.l. (Dodson, 2002; Telenius and Shah, 2016) and individuals of *E. macrum* in Zamora-Chinchipe province between 1000 and 1100 m.a.s.l. (Magill et al., 2016). Both species are naturally distributed in southern Ecuador but thus far have never been recorded in the RBSF.

### Seedling-Trap Experiment

**FIGURE 1:**
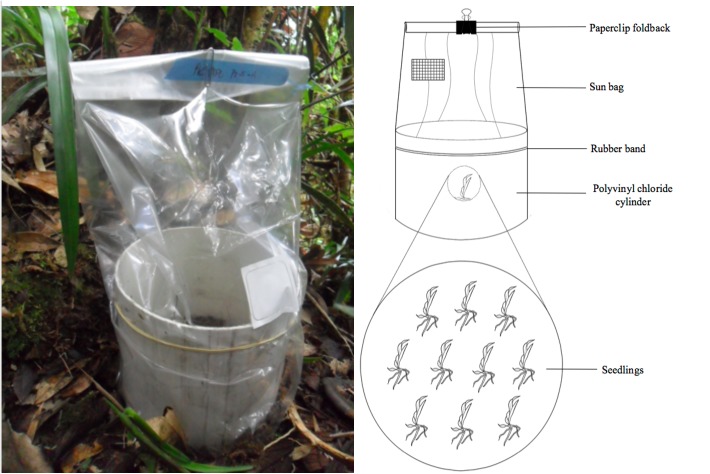
Photo and schematic illustration of the seedling-trap systems. Seedling-traps included polyvinyl chloride cylinder, 10 seedlings, covered with a sun bag adjusted with a rubber band and a paperclip foldback.

**FIGURE 2:**
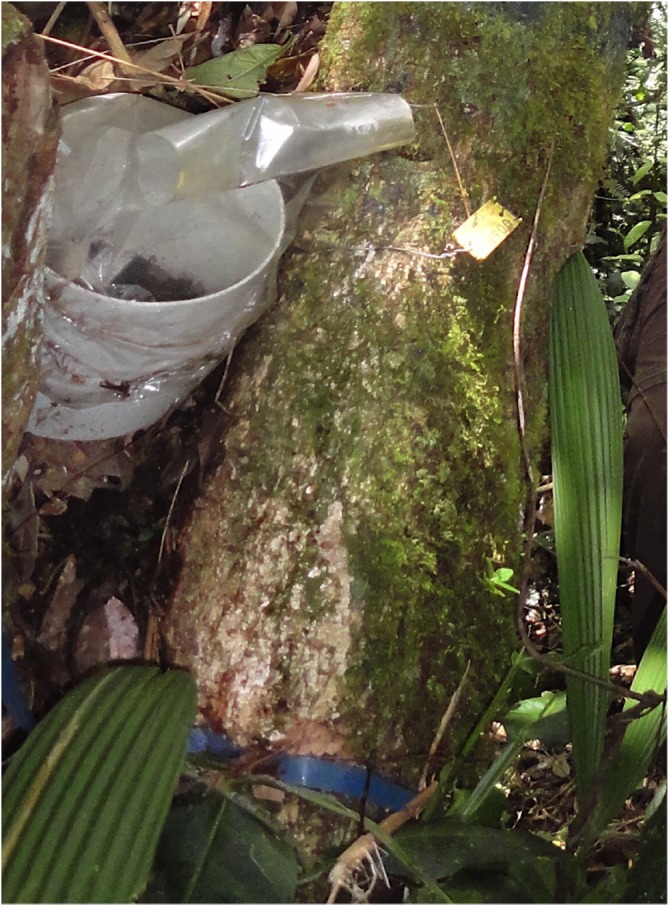
Set-up of a seedling-trap system on a tree branch.

Seedling-trap systems consisted of a polyvinyl chloride cylinder (20 cm diameter and 15 cm height) with 10 orchid seedlings inside (same species) and covered with a 0.02 μm pore size sun bag (Sigma-Aldrich, St. Louis, Missouri, United States) to avoid herbivory and litter input (Figure 1). The seedling-trap systems were placed in direct contact with tree branches (Figure 2). Only tree branches supporting at least one orchid naturally established were selected. No particular attention was given to the height of the tree branches. In both transects (T2 and Q5), seedling-traps were installed along 10 elevational points, separated by ∼30 m.a.s.l. along a range between 1850 m.a.s.l. and 2150 m.a.s.l. (assigned as A1 to A10). For each elevational point, one seedling-trap from *C. retusum* and one from *E. macrum* were established. All seedling-traps installed along one transect and harboring the same orchid species were considered as one treatment that evaluated a factor, resulting in four treatments. The four treatments included the evaluation of the effect of factors such as orchid species, site, altitude and temporal variation on the composition of mycorrhizal communities. In total, 40 seedling-trap systems (2 transect × 10 elevational levels × 2 orchid species) were established that included 10 replicates per treatment.

Seedling-trap systems were sampled 3 (S1) and 12 (S2) months after their installation. The number of seedlings collected from each seedling-trap at 3 and 12 months varied between 1 and 4, depending on the number of surviving seedlings per system. In both samplings, all the roots collected from the plants in each seedling-trap system were pooled as a single sample and stored in 70% ethanol for molecular analysis. To confirm mycorrhizal colonization, cross-sections of three randomly selected samples were stained with methyl blue solution (0.05%, Merck, Darmstadt, Germany) for 3 min. and microscopically checked for the presence of pelotons.

### DNA Sequencing

Total DNA from seedling roots was extracted using the PureLink^®^ Genomic Plant DNA Purification Kit (Invitrogen) according to the manufacturer’s protocol. To analyze the internal transcribed spacer 2 (ITS2) region of nuclear ribosomal DNA (nrDNA), two primer pairs were used for PCR amplification (see Waud et al., 2014): ITS86F (Turenne et al., 1999) combined with ITS4 (White et al., 1990); and ITS3 (White et al., 1990) combined with ITS4 (White et al., 1990). PCR amplifications were performed in 20 μl reaction volume containing 4 μl of 5X Phusion HF Buffer, 0.4 μl of dNTPs (10 mM), 0.4 μl of each primer, 0.8 μl BSA 10%, 0.2 μl of Phusion DNA polymerase (Thermo Fisher Scientific), 11.8 μl of ultrapure water and 2 μl of total DNA. PCR conditions for both primers combinations were as follows: initial denaturation at 98°C for 30 s followed by 35 cycles of denaturation at 98°C for 10 s, annealing at 60°C for 20 s, extension at 72°C for 30 s and a final extension at 72°C for 10 min. After resolving the amplicons by agarose gel electrophoresis, only amplicons within the expected size range (∼250–350 bp) were kept. Amplicons obtained from the same DNA template with different primer pairs were mixed as a single sample and purified using the Wizard^®^ SV Gel and PCR Clean-Up System (Promega, United States). The quality of the purified DNA amplicons was determined through the evaluation of the AD260/280 ratio calculated using the Spectrophotometer NanoDrop^®^ 2000c (Thermo Scientific, Wilmington, Delaware, United States). Finally, sequencing was performed using the MiSeq Illumina platform at IMGM Laboratories GmbH (Martinsried, Germany).

### Sequence Analysis

Raw Illumina sequence data were processed using the UPARSE software (Edgar, 2013). First, the overlapping paired reads were assembled into single sequences with the “fastq_mergepairs” command. In addition, the –fastq_nostagger option was used to discard staggered pairs. Next, quality filter was applied using the “fastq_filter” command with a maximum expected error threshold of 0.3 for single sequences. To discard sequences of short length, the truncation length was set to 240 bp. Singletons were discarded with the “derep_fulllength” command. Operational taxonomic units (OTUs) were determined using the “cluster_otus” command, and the sequences displaying 97% homology were classified in the same OTU. The OTUs were further assigned taxonomic identities to the highest taxonomic rank possible with the BLASTN algorithm implemented in UNITE database^1^; (Abarenkov et al., 2010a) through the PlutoF (Abarenkov et al., 2010b) web-based sequence management workbench (2017-09-09 release), including uncultured/environmental entries. Finally, OTUs with taxonomic identity attributed to a member of OMF (Suárez et al., 2006, 2008; Kottke et al., 2010; Valadares et al., 2015) were retained for further analyses. Sequences were submitted to GenBank under the accession number PRJNA396957.

### Statistical Analysis

The OMF-OTUs frequency of occurrence was transformed into binary matrix on a per-sample basis as input data to make inferences on OMF richness and community composition as a function of the orchid host, sites, elevational levels or the temporal variation. In the matrix, the columns corresponding to each of the recovered seedling-trap systems were codified as follows: transect name, orchid species, sampling time and elevational level (T2 = transect T2, Q5 = transect Q5; C = *C. retusum*, E = *E. macrum*; S1 = first sampling and S2 = second sampling and A1 – A10 = elevational levels) i.e., T2CS1_A1, T2ES1_A1, Q5CS1_A1, Q5ES1_A1 and so on. To evaluate the entire OMF richness per treatment, the presence/absence matrix was used as input data for the construction of accumulation curves (Jiménez-Valverde and Hortal, 2003) applying a sample-based rarefaction method with 100 permutations, implemented in EstimateS 9.1.1 (Colwell, 2013; Orgiazzi et al., 2013; Senés-Guerrero et al., 2014). The seedling-trap systems from Q5ES1 and Q5CS2 were not considered for statistical analysis due to low number of samples recovered (two seedling-traps each).

To test differences in OMF communities among co-existing orchid species (T2CS1 vs. T2ES1) and temporal variation (T2CS1 vs. T2CS2), individually per each elevational level, beta diversity determined as the ratio of the number of OMF-OTUs shared and the total number of OTUs in samples (Orgiazzi et al., 2013) was calculated by similarity indices of Chao-Jaccard and Chao-Sørensen using Estimates 9.1.1 software (Colwell, 2013). Samples from each elevational level were considered as a replicate. Similarly, to evaluate the effect of the elevation on the mycorrhizal community per treatment (T2CS1, T2ES1, T2CS2, and Q5CS1), the similarity was calculated by pairwise comparison between elevational levels of the same treatment.

Permutational analysis of variance (Permanova) was performed with 999 permutations using the adonis function in the Vegan package (Oksanen et al., 2016) of R (R Core Team, 2015) to evaluate whether OMF communities differed significantly as a function of host phylogeny and the sites of orchid occurrence. Finally, to visualize the differences among sites (T2CS1 vs. Q5CS1) and temporal variation (T2CS1 vs. T2CS2) a non-metric multidimensional scaling (NMDS) was constructed using SPSS 22 (IBM Corp., Somers, NY, United States). The Jaccard coefficient was used as a distance measure and each elevational level as a replicate.

In order to evaluate if the sequencing depth differences between samples impact the statistical findings, the sequence reads per sample were rarefied to the 10% of the highest sequence reads per samples using Seqtk software (Supplementary Materials). The sequence analysis and the statistical analysis were performed as was mentioned above.

## Results

In the first sampling (i.e., at month 3), nine seedling-trap systems were recovered from each treatment: T2CS1, T2ES1, and Q5CS1. The two seedling-trap systems recovered from Q5ES1 were not considered due to low number of samples. In the second sampling (i.e., at month 12), samples from eight seedling-trap systems were recovered from T2CS2, and, as in the first sampling, Q5CS2 was not considered due to low number of samples (Supplementary Table 1).

### Fungal OTUs

**FIGURE 3:**
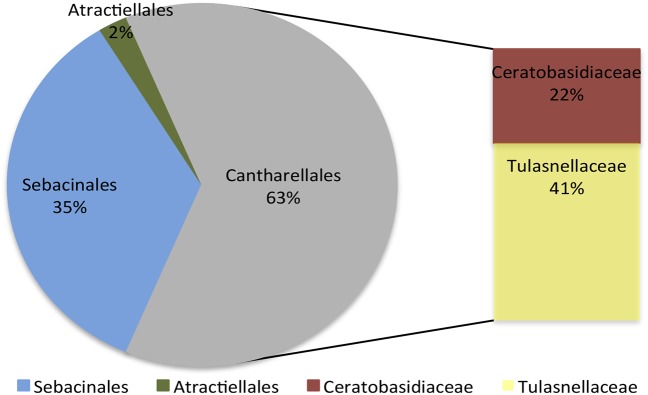
Frequency distribution of the identified fungal orders of the orchid mycorrhizal fungi identified in association with *Cyrtochilum retusum* and *Epidendrum macrum*.

In the 35 samples assessed, between 24,385 and up to 249,004 reads per sample were obtained. In total, 865,071 high-quality sequences of ITS2 (∼300 bp) were retrieved after chimeric sequences were discarded (11.2% of total number of sequences). Operational taxonomic unit reconstruction based on a 97% sequence similarity cutoff resulted in 1757 fungal OTUs. BLAST analysis of the representative sequences from reconstructed OTUs showed the presence of sequences matching both mycorrhizal and non-mycorrhizal fungi; the latter were not considered in subsequent analyses. Putative mycorrhizal fungi were ascribed to 83 OTUs belonging to Cantharellales (53 OTUs), Sebacinales (28 OTUs) and Atractiellales (2 OTUs) orders (Figure 3 and Supplementary Table 2).

**FIGURE 4:**
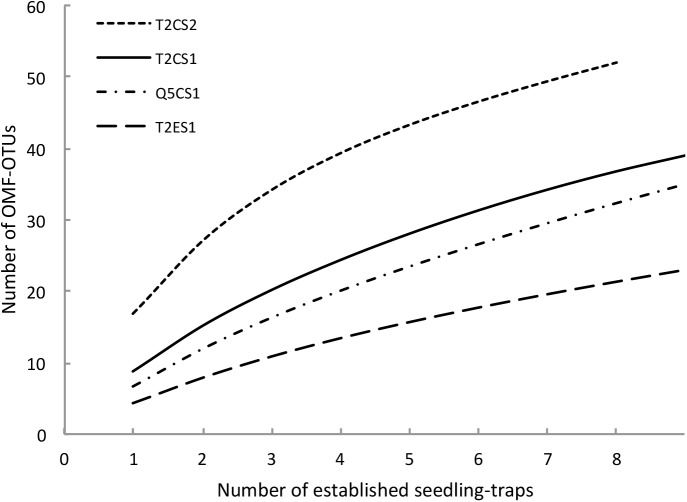
Rarefaction curves of orchid mycorrhizal fungi OTU (operational taxonomic unit) richness in four treatments of field experiment. T2: transect T2; Q5: transect Q5; C: *Cyrtochilum retusum*; E: *Epidendrum macrum*; S1: 1^st^ sampling and S2: 2^nd^ sampling.

Rarefaction curves of OMF-OTUs did not reach an asymptote in any of the treatments (Figure 4). In total, 39, 23, 35 and 52 OTUs were found in the T2CS1, T2ES1, Q5CS1, and T2CS2 treatments, respectively. Six OTUs (OTU19, OTU24, OTU144, OTU225, OTU358, and OTU4582) were identified in all treatments in at least one elevational level. Only OTU138 was identified at all elevational levels in the treatment T2CS2.

### Mycorrhizal Communities as a Function of the Orchid Species, Site, Elevation and Temporal Variation

Mycorrhizal communities evaluated as a function of the orchid species (T2CS1 vs. T2ES1) showed that *C. retusum* had a greater number of OMF-OTUs than *E. macrum*. Chao-Jaccard and Chao-Sørensen indices revealed low similarity in the composition of OMF communities between *C. retusum* and *E. macrum* (Table 1). Permanova analysis performed to contrast the richness among co-existing orchid species showed significant difference in the OMF communities (*P*-value = 0.046).

**Table 1:** Number of orchid mycorrhizal fungi (OMF) OTUs and the similarity indices for the co-occurring orchids *Cyrtochilum retusum* and *Epidendrum macrum* at the different elevational levels (A1–A10).

Elevational level	OMF-OTUs from *C. retusum*	OMF-OTUs from *E. macrum*	Shared OMF-OTUs	Chao-Jaccard	Chao-Sorensen
A1	6	2	0	0	0
A2	7	2	2	0.286	0.444
A4	7	2	1	0.125	0.222
A5	10	3	2	0.182	0.308
A6	18	4	2	0.1	0.182
A8	10	7	1	0.063	0.118
A9	6	5	3	0.375	0.545
A10	2	3	0	0	0


**FIGURE 5:**
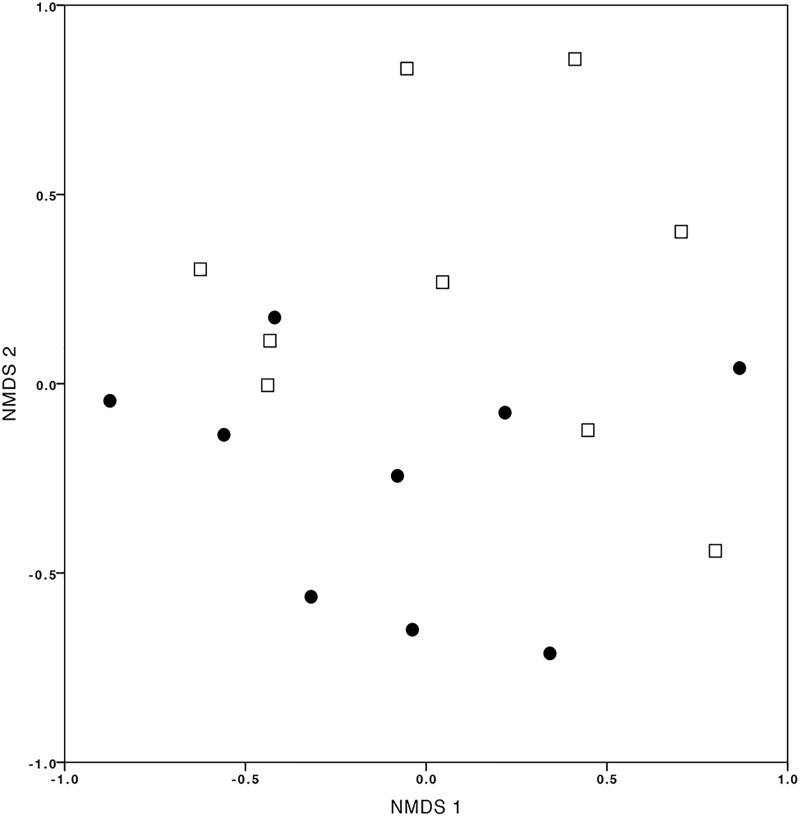
Non-multidimensional scaling (NMDS) plot of orchid mycorrhizal fungal communities associated with *Cyrtochilum retusum* at T2 (black dots) and Q5 (white squares) sites (stress value = 0.070).

Assessing the influence of the site on OMF communities (T2CS1 vs. Q5CS1), the NMDS ordination showed different OMF communities but with a substantial overlap (Figure 5). In total, 39 and 35 OTUs were identified in treatments T2CS1 and Q5CS1, respectively, and from these, 17 OMF-OTUs overlapped at both sites. Analysis by Permanova provided evidence to confirm that the community composition of OMF associated with *C. retusum* was not significantly different among the sites (*P-*value = 0.242).

Moreover, along elevational levels the Chao-Sorensen and Chao-Jaccard indices, calculated independently for each treatment (T2CS1, T2ES1, and Q5CS1), showed low similarity in the composition of OMF communities in almost all the pairwise comparisons. The elevational levels with greater similarity of the OMF communities were not necessarily the closest (Supplementary Table 3).

**FIGURE 6:**
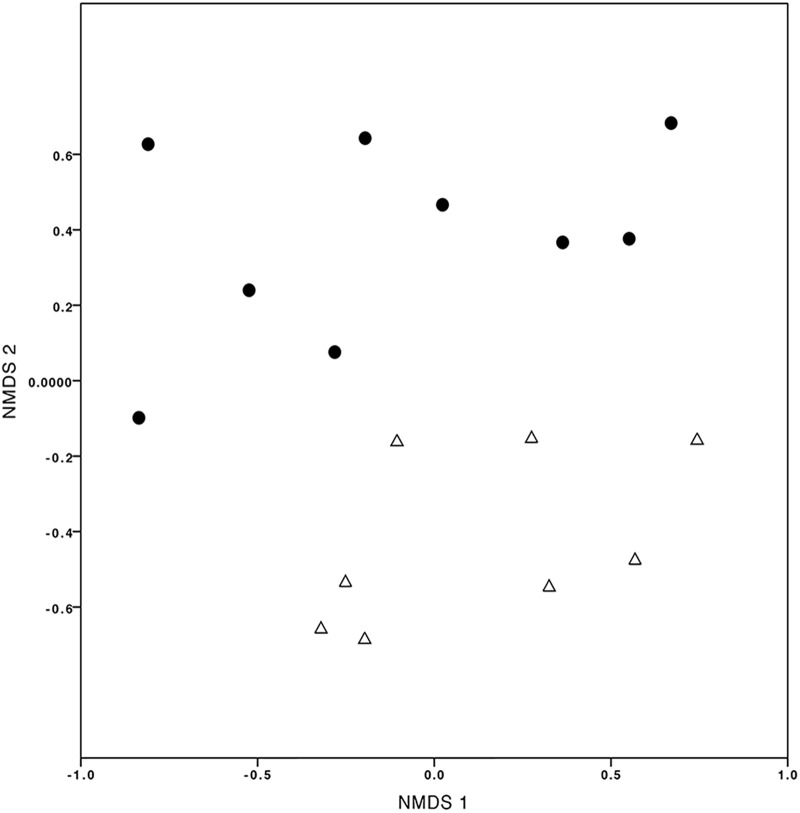
Non-multidimensional scaling (NMDS) plot of mycorrhizal fungi detected in *Cyrtochilum macrum* sampled at two colonization times. The black dots correspond to samples obtained from the first sampling (T2CS1 treatment) and the white triangles correspond to the second sampling (T2CS2 treatment), after three months and one year, respectively, of the assay was established (stress value = 0.058).

Finally, evaluating the temporal variation (T2CS1 vs. T2CS2), the number of OMF-OTUs detected per seedling-trap system varied between 6 and 18 after three months (1st sampling, T2CS1) and between 5 and 22 after 12 months (2nd sampling, T2CS2) (Supplementary Table 4). Chao-Sorensen and Chao-Jaccard indices calculated by contrasting T2CS1 and T2CS2, independently per each elevational level, showed low similarity in the OMF communities (Supplementary Table 4), as evident in the NMDS (Figure 6).

Contrasting the analyses outputs between samples rarefied to the 10% of the highest sequence reads per sample and samples non-rarefied, similar patterns of OMF-OTUs richness and OMF community composition were observed (Supplementary Materials).

## Discussion

### Mycorrhizal Fungal Community Composition

In this study, the OMF communities associated with the epiphytic orchids *C. retusum* and *E. macrum*, established along an elevation level, were assessed in the RBSF (southern Ecuador) using a seedling-trap experiment. Although field experiments are infrequently performed, they represent a more realistic approach to evaluate the interaction between orchids and fungi. For instance, with the use of seed packets (field experiments) it was demonstrated that habitat conditions had little influence on seed germination of four *Epipactis* species (Těšitelová et al., 2012). Moreover, several studies that evaluated OMF associated with orchids at different developmental stages demonstrated the occurrence of different OMF communities across orchid lifecycle (Oja et al., 2017; Waud et al., 2017).

In accordance with previous studies performed in tropical areas (Kottke et al., 2010; Cevallos et al., 2017), the present results revealed that epiphytic orchids were associated with a highly diverse group of mycorrhizal fungi (represented by 83 OTUs), comprising members of Tulasnellaceae, Ceratobasidiaceae, Serendipitaceae and Atractiellales. Members of Tulasnellaceae appear to be globally distributed, as they have been frequently reported in association with epiphytic orchids in many forests (Martos et al., 2012; Riofrío et al., 2013; Suárez et al., 2016; Oberwinkler et al., 2017). Consistently, in the present study the dominant fungal group associated with *C. retusum* and *E. macrum* was Tulasnellaceae, representing 41% of the entire mycorrhizal fungi identified. Serendipitaceae was the second most frequent group identified (35%), supporting earlier studies that recognized Serendipitaceae as a persistent orchid partner worldwide (Suárez et al., 2008; Martos et al., 2012). In a recent study performed in the same geographical location (Cevallos et al., 2017), fungi putatively assigned to Serendipitaceae were the most frequent taxa identified in association with epiphytic adult orchids of the Cymbidieae tribe. However, here we added a second set of complementary primers (ITS86F/ITS4 combined with ITS3/ITS4) to reduce the biases and reach a more accurate description of fungal communities (Waud et al., 2014).

Six OMF-OTUs (OTU19, OTU24, OTU144, OTU225, OTU358, and OTU4582) were detected in all treatments. Among these, OTU19 (Ceratobasidiaceae), OTU24 (Serendipitaceae) and OTU144 (Serendipitaceae) were phylogenetically congruent (≥ 97%) with OTU33 (Ceratobasidium), OTU2 (Serendipitaceae) and OTU13 (Serendipitaceae), respectively, reported by Cevallos et al. (2017). These OTUs identified by Cevallos et al. (2017) were broadly identified in two populations of *Cyrtochilum flexuosum, C. myanthum* and *Maxillaria calantha*, distributed in the surrounding areas of Podocarpus National Park. This finding supports the hypothesis that a core of generalist fungi with wide distribution could be an essential component of the OMF communities (Pandey et al., 2013; Xing et al., 2017).

### Effect of Orchid Species, Site, Elevation and Temporal Variation on Mycorrhizal Fungal Community Composition

The exact factors driving variation in the composition and structure of OMF communities are still unclear (Jacquemyn et al., 2016). There is growing evidence that mycorrhizal variation is influenced by such factors as seasonal dynamics (Oja et al., 2015), biotope (Han et al., 2016), orchid species (Jacquemyn et al., 2010) or orchid life cycle (Bidartondo and Read, 2008). The present results seem to confirm these observations because clear differences in OMF communities were noticed between co-existing orchid species, along an elevational gradient and during a temporal variation.

The different OMF communities observed between co-existing orchid species strongly support previous results showing divergent mycorrhizal communities associated with terrestrial orchids from the genera *Anacamptis, Neotinea, Orchis, Ophrys* and *Serapias* co-occurring at a given site (Jacquemyn et al., 2014). Theoretically, two species are not able to co-exist when they are using the same resources (Tilman, 1982) unless there is small-scale habitat heterogeneity that allows niche differentiation, for instance, segregation of mycorrhizal fungi (Jacquemyn et al., 2014; Voyron et al., 2017). The preference for different mycorrhizal fungal communities might represent a niche partition that contributes to orchid co-existence (McCormick and Jacquemyn, 2014). More specifically, different OMF communities potentially shape the realized niche among inhabiting species and might have considerable implications on the dynamics of orchid communities as a result of limiting factors of the habitat (Gerz et al., 2018). The mycorrhizal partners associated with each orchid species might represent part of an ancestral niche prevalent throughout co-evolutionary processes (Losos, 2008; Selosse, 2014). Therefore, it is suggested that although each orchid species is not associated with a specific set of mycorrhizal fungi, the niche-partitioning and the orchid-fungi co-evolution may determine the fungi effectively associated with a particular orchid species.

Similarly to orchid species, the site of orchid occurrence has been frequently reported as a determining factor of OMF communities (Xing et al., 2013; Cevallos et al., 2017). Here we found no significant differences in OMF community composition between the two study sites (transect T2 and Q5). Contrasting results have been reported by Kartzinel et al. (2013), who identified different mycorrhizal communities across 11 populations of *E. firmum*, an epiphytic orchid naturally distributed in Costa Rica. These differences were mostly attributed to the divergent climatic and geographic conditions of the study sites. Moreover, different OMF communities have also been observed in association with the terrestrial orchid *Anacamptis morio*, suggesting an effect of the environmental variation (Voyron et al., 2017). The similarity between the OMF communities of the two study sites (i.e., T2 vs. Q5) may be due in part to the similarity of the sites environmental characteristics (i.e., rainfall, temperature), despite floristic differences between ridge and ravine forest (Homeier et al., 2008). Identical results were obtained in a recent study evaluating OMF associated with epiphytic orchids distributed in four sites of the Cajas National Park (Herrera et al., in press).

The OMF communities associated with *C. retusum* and *E. macrum* consisted of a stable component that include keystone species recorded in both site and a dynamic component comprising a number of fungi exclusively identified at specific site. Similar results were observed in two populations of *Dendrobium officinale*, in which the OMF communities included a set of fungi widely identified across orchid populations and a set of fungi specifically identified at one of the two orchid populations but not both (Xing et al., 2013). Overall, these findings support the hypothesis that OMF communities are site-adjusted around keystone fungal species that are widely distributed, as was observed in the OMF communities associated with *C. flexuosum, C. myanthum* and *M. calantha* (Cevallos et al., 2017). However, further analyses that include more sites are needed to confirm this hypothesis.

It is generally accepted that elevation is a factor that drives the structure of communities (i.e., plants and animals) (McCain, 2005; Grytnes and Beaman, 2006). However, the effect of elevation on OMF communities is virtually unknown. Suárez et al. (2006) reported a decrease of the OMF richness with increasing elevation but in the present study, the fungal richness did not always decrease with increasing elevation. Although *C. retusum* and *E. macrum* have not been recorded in our study sites (but reported in southern Ecuador), we suggest that the identified fungi could represent a potential mycorrhizal niche. The contrast between orchid mycorrhizal communities showed significant differences along the elevational level, however, there was not a clear pattern of the mycorrhizal fungi communities at the elevation level and moreover the limited number of replicates per elevational level made it impossible to make further ecological inferences. It still remains to be elucidated whether the changes in the OMF detected along the elevational gradient are due to an opportunistic association (Dearnaley et al., 2012) or if the elevation induced changes in the OMF community.

In addition, the present results clearly show a significant temporal variation in the composition of OMF communities. Indeed, OTUs-OMF richness increased across time. Changes on mycorrhizal communities have been observed in relation to the development stages of hosts and season (Bidartondo and Read, 2008; Kohout et al., 2013; Ercole et al., 2015; Oja et al., 2015). For instance, Bidartondo and Read (2008) reported that *Cephalanthera damasonium* and *Cephalanthera longifolia* had different OMF communities across orchid development but with a subset of fungi identified at all orchid life stages. Further long-term studies are necessary to validate if these differences in OMF communities are due to the orchid developmental stage and the concomitant changes in physiological processes (i.e., distinctive nutrient uptake) (Rasmussen and Rasmussen, 2009; Kohout et al., 2013).

We suggest that OMF communities experience a successional process of colonization along the development stages of the plant, as the results show that the OMF communities were structured by keystone species (OMF identified either after three or 12 months) and a dynamic component (OMF changing over time). *In vitro* experiments have demonstrated that keystone species of OMF that were isolated from adult plants were able to promote orchid seed germination (Fracchia et al., 2013). Furthermore, in field experiments, orchids at early developmental stages were abled to form associations with a set of available OMF (Bidartondo and Read, 2008), but these OMF may not necessarily be present in the roots of the orchid throughout its entire life cycle (Shimura et al., 2009). Information on the temporal dynamics of OMF communities is currently limited (Ercole et al., 2015), and periodic samplings across the entire orchid life cycle is required to determine if keystone OMF remain throughout orchid development. It is proposed that the OMF keystone species identified here represent a permanent core of OMF, as they were found across sampling sites and orchid species (Cevallos et al., 2017).

## Conclusion

The present study shows that the combination of orchid seedling-trap experiments and Illumina MiSeq ITS2 sequencing analysis is an adequate approach for determining the composition of OMF communities and the biotic and abiotic factors that structure those communities. The results support the observation that OMF communities are composed of a dynamic and a core set of keystone species and that the orchid-fungi symbiosis is a dynamic interaction governed by temporal variations, environmental filtering and co-evolutionary history.

## Author Contributions

SC and JS designed the experiments and conducted the fieldwork. SC performed the molecular and statistical analyses. SC, SD, and JS wrote the manuscript.

## Conflict of Interest Statement

The authors declare that the research was conducted in the absence of any commercial or financial relationships that could be construed as a potential conflict of interest.

## Footnotes

1 http://unite.ut.ee
