# Supplementary material for: In situ Orchid Seedling-Trap Experiment Shows Few Keystone and Many Randomly Associated Mycorrhizal Fungal Species During Early Plant Colonization
Source: Front Plant Sci. 2018 Nov 16;9:1664. doi: 10.3389/fpls.2018.01664 (PMC6250785; doi:10.3389/fpls.2018.01664)
Supplement: Supplementary file 1 [file Table_1.DOCX]

Supplementary Material

*In Situ* Orchid Seedling-Trap Experiment Shows Few Keystone and Many Randomly-Associated Mycorrhizal Fungal Species During Early Plant Colonization

Stefania Cevallos, Stéphane Declerck, Juan Pablo Suárez *

*** Correspondence:** Juan Pablo Suárez: jpsuarez@utpl.edu.ec

**Supplementary Table 1** Collected seedling-traps and number of identified OTUs. T2: Transect T2, Q5: Transect Q5; C: *Cyrtochilum retusum,* E: *Epidendrum macrum*; S1: 1^st^ sampling, S2: 2^nd^ sampling and A1-A10: Altitudinal levels.

| **Sampled seedling-trap** | **Mycorrhizal OTUs** |  | **Non-mycorrhizal OTUs** |
| --- | --- | --- | --- |
| T2CS1_A1 | 6 |  | 63 |
| T2CS1_A2 | 7 |  | 56 |
| T2CS1_A3 | - |  | - |
| T2CS1_A4 | 7 |  | 76 |
| T2CS1_A5 | 10 |  | 88 |
| T2CS1_A6 | 18 |  | 99 |
| T2CS1_A7 | 8 |  | 152 |
| T2CS1_A8 | 10 |  | 144 |
| T2CS1_A9 | 11 |  | 73 |
| T2CS1_A10 | 2 |  | 52 |
| T2ES1_ A1 | 2 |  | 107 |
| T2ES1_ A2 | 2 |  | 72 |
| T2ES1_ A3 | 3 |  | 68 |
| T2ES1_ A4 | 2 |  | 32 |
| T2ES1_ A5 | 3 |  | 99 |
| T2ES1_ A6 | 4 |  | 33 |
| T2ES1_A7 | - |  | - |
| T2ES1_A8 | 7 |  | 34 |
| T2ES1_ A9 | 13 |  | 77 |
| T2ES1_ A10 | 3 |  | 56 |
| Q5CS1_ A1 | - |  | - |
| Q5CS1_ A2 | 5 |  | 46 |
| Q5CS1_ A3 | 4 |  | 92 |
| Q5CS1_ A4 | 21 |  | 161 |
| Q5CS1_ A5 | 5 |  | 91 |
| Q5CS1_ A6 | 5 |  | 69 |
| Q5CS1_ A7 | 2 |  | 74 |
| Q5CS1_ A8 | 8 |  | 131 |
| Q5CS1_ A9 | 5 |  | 85 |
| Q5CS1_ A10 | 5 |  | 69 |
| T2CS2_ A1 | 14 |  | 148 |
| T2CS2_ A2 | 5 |  | 136 |
| T2CS2_ A3 | - |  | - |
| T2CS2_ A4 | 11 |  | 163 |
| T2CS2_ A5 | 22 |  | 114 |
| T2CS2_ A6 | 19 |  | 228 |
| T2CS2_ A7 | 19 |  | 111 |
| T2CS2_ A8 | - |  | - |
| T2CS2_ A9 | 25 |  | 140 |
| T2CS2_ A10 | 19 |  | 150 |
