# Supplementary material for: In situ Orchid Seedling-Trap Experiment Shows Few Keystone and Many Randomly Associated Mycorrhizal Fungal Species During Early Plant Colonization
Source: Front Plant Sci. 2018 Nov 16;9:1664. doi: 10.3389/fpls.2018.01664 (PMC6250785; doi:10.3389/fpls.2018.01664)
Supplement: Supplementary file 2 [file Table_2.DOCX]

Supplementary Material

*In Situ* Orchid Seedling-Trap Experiment Shows Few Keystone and Many Randomly-Associated Mycorrhizal Fungal Species During Early Plant Colonization

Stefania Cevallos, Stéphane Declerck, Juan Pablo Suárez *

*** Correspondence:** Juan Pablo Suárez: jpsuarez@utpl.edu.ec

**Supplementary Table 2** Operational taxonomic units putatively assigned to mycorrhizal fungi using UNITE database. * sequences identity lower than 90%.

| OTU Id | Number of sequences | Order | Family/Genus | Length (bp) | Score | E-value | Sequence identity % |
| --- | --- | --- | --- | --- | --- | --- | --- |
| OTU_19 | 26844 | Cantharellales | Ceratobasidiaceae | 360 | 568.1 | 0 | 99.12 |
| OTU_24 | 3256 | Sebacinales | - | 329 | 413.6 | 0 | 89.86 |
| OTU_27 | 8757 | Cantharellales | Cantharellales | 362 | 523.5 | 0 | 94.25 |
| OTU_34 | 7526 | Sebacinales | Serendipitaceae | 317 | 509.7 | 0 | 97.79 |
| OTU_46 | 6628 | Cantharellales | Ceratobasidium | 443 | 652.2 | 0 | 94.87 |
| OTU_60 | 2186 | Sebacinales | - | 348 | 468.5 | 0 | 92.57 |
| OTU_138 | 2023 | Cantharellales | - | 382 | 152.6 | 0 | 74.41 |
| OTU_144 | 293 | Sebacinales | - | 335 | 566.4 | 0 | 99.4 |
| OTU_153 | 1524 | Sebacinales | - | 336 | 477.1 | 0 | 94.07 |
| OTU_178 | 370 | Cantharellales | - | 347 | 94.3 | 0 | 92.75 |
| OTU_191 | 1051 | Cantharellales | Ceratobasidium | 428 | 638.5 | 0 | 95.56 |
| OTU_196 | 376 | Sebacinales | - | 341 | 506.3 | 0 | 95.56 |
| OTU_204 | 474 | Sebacinales | Serendipita | 459 | 130.3 | 0 | 94.44 |
| OTU_210 | 409 | Sebacinales | - | 395 | 612.7 | 0 | 94.44 |
| OTU_223 | 1138 | Cantharellales | - | 410 | 595.6 | 0 | 94.87 |
| OTU_225 | 4722 | Cantharellales | Ceratobasidiaceae | 353 | 542.4 | 0 | 99.38 |
| OTU_298 | 406 | Sebacinales | - | 328 | 509.7 | 0 | 96.65 |
| OTU_325* | 167 | Cantharellales | - | 383 | 116.6 | 0 | 72.51 |
| OTU_358 | 102 | Cantharellales | - | 331 | 80.5 | 0 | 91.8 |
| OTU_402 | 131 | Sebacinales | - | 334 | 549.2 | 0 | 98.5 |
| OTU_418 | 95 | Atractiellales | - | 354 | 609.3 | 0 | 100 |
| OTU_426 | 98 | Cantharellales | Ceratobasidium | 363 | 559.5 | 0 | 96.43 |
| OTU_473 | 75 | Cantharellales | Ceratobasidium | 358 | 502.9 | 0 | 93.85 |
| OTU_492 | 53 | Sebacinales | - | 334 | 569.8 | 0 | 99.7 |
| OTU_548 | 51 | Sebacinales | - | 340 | 544.1 | 0 | 97.65 |
| OTU_566 | 138 | Sebacinales | Sebacinaceae | 339 | 521.7 | 0 | 96.46 |
| OTU_579 | 178 | Cantharellales | - | 403 | 638.5 | 0 | 97.51 |
| OTU_634 | 147 | Cantharellales | - | 358 | 478.8 | 0 | 95.62 |
| OTU_699 | 215 | Cantharellales | - | 347 | 94.3 | 0 | 92.75 |
| OTU_715* | 49 | Cantharellales | - | 383 | 84 | 0 | 70.83 |
| OTU_763 | 70 | Cantharellales | Ceratobasidiaceae | 357 | 542.4 | 0 | 96.08 |
| OTU_830 | 57 | Cantharellales | Ceratobasidium | 416 | 700.3 | 0 | 99.28 |
| OTU_907* | 51 | Cantharellales | - | 381 | 99.4 | 0 | 71.69 |
| OTU_933 | 205 | Cantharellales | Ceratobasidium | 364 | 569.8 | 0 | 96.98 |
| OTU_1047 | 34 | Sebacinales | - | 384 | 587 | 0 | 97.81 |
| OTU_1089 | 20 | Sebacinales | - | 334 | 494.3 | 0 | 96.01 |
| OTU_1103* | 194 | Cantharellales | - | 382 | 128.6 | 0 | 73.21 |
| OTU_1168 | 39 | Sebacinales | - | 383 | 518.3 | 0 | 92.53 |
| OTU_1176 | 65 | Cantharellales | - | 331 | 90.8 | 0 | 95.08 |
| OTU_1226 | 15 | Atractiellales | - | 346 | 585.3 | 0 | 99.42 |
| OTU_1259 | 90 | Cantharellales | Ceratobasidium | 411 | 650.5 | 0 | 97.32 |
| OTU_1305* | 114 | Cantharellales | - | 382 | 111.4 | 0 | 72.34 |
| OTU_1308* | 41 | Sebacinales | Sebacinaceae | 427 | 473.7 | 0 | 87.94 |
| OTU_1408* | 27 | Sebacinales | Sebacinaceae | 339 | 265.9 | 0 | 81.38 |
| OTU_1410 | 24 | Sebacinales | - | 340 | 580.1 | 0 | 99.71 |
| OTU_1417* | 6 | Cantharellales | - | 309 | 326 | 0 | 85.25 |
| OTU_1442* | 128 | Cantharellales | - | 382 | 138.9 | 0 | 73.74 |
| OTU_1451* | 22 | Cantharellales | - | 382 | 104.6 | 0 | 76.33 |
| OTU_1470 | 41 | Sebacinales | - | 366 | 609.3 | 0 | 98.91 |
| OTU_1527 | 35 | Cantharellales | - | 334 | 554.4 | 0 | 98.8 |
| OTU_1592* | 101 | Cantharellales | - | 383 | 108 | 0 | 72.15 |
| OTU_1628 | 25 | Cantharellales | - | 382 | 89.1 | 0 | 91.3 |
| OTU_1863 | 36 | Cantharellales | - | 330 | 85.7 | 0 | 93.44 |
| OTU_1912 | 25 | Cantharellales | Ceratobasidium | 445 | 743.2 | 0 | 98.88 |
| OTU_2038* | 14 | Cantharellales | - | 433 | 401.6 | 0 | 87.6 |
| OTU_2180 | 4 | Cantharellales | Ceratobasidiaceae | 358 | 585.3 | 0 | 98.32 |
| OTU_2416* | 63 | Cantharellales | - | 405 | 118.3 | 0 | 72.32 |
| OTU_2462 | 132 | Cantharellales | - | 370 | 116.6 | 0 | 91.21 |
| OTU_2514* | 6 | Cantharellales | Tulasnellaceae | 431 | 398.1 | 0 | 84.13 |
| OTU_2661* | 13 | Cantharellales | - | 382 | 109.7 | 0 | 72.22 |
| OTU_2870 | 37 | Cantharellales | - | 340 | 94.3 | 0 | 92.75 |
| OTU_2877* | 8 | Cantharellales | - | 382 | 111.4 | 0 | 72.3 |
| OTU_3176 | 5 | Sebacinales | - | 371 | 581.8 | 0 | 97.04 |
| OTU_3440 | 2 | Sebacinales | Serendipitaceae | 317 | 417 | 0 | 92.11 |
| OTU_3477 | 6 | Sebacinales | - | 364 | 513.2 | 0 | 93.73 |
| OTU_3539 | 7 | Cantharellales | Ceratobasidiaceae | 390 | 604.2 | 0 | 96.67 |
| OTU_3753* | 2 | Cantharellales | Ceratobasidium | 370 | 343.2 | 0 | 84.31 |
| OTU_3843* | 6 | Cantharellales | - | 436 | 278 | 0 | 81.02 |
| OTU_3866 | 80 | Cantharellales | - | 340 | 94.3 | 0 | 92.75 |
| OTU_3927 | 2 | Cantharellales | - | 416 | 616.2 | 0 | 94.99 |
| OTU_3928 | 3 | Sebacinales | Serendipitaceae | 386 | 576.7 | 0 | 95.6 |
| OTU_4003 | 2 | Cantharellales | Ceratobasidiaceae | 445 | 611 | 0 | 93.26 |
| OTU_4038 | 2 | Cantharellales | - | 469 | 604.2 | 0 | 91.51 |
| OTU_4049* | 5 | Cantharellales | Ceratobasidiaceae | 395 | 372.4 | 0 | 84.58 |
| OTU_4105 | 8 | Cantharellales | Ceratobasidiaceae | 445 | 623 | 0 | 93.72 |
| OTU_4199 | 8 | Cantharellales | - | 328 | 535.5 | 0 | 99.37 |
| OTU_4217 | 3 | Cantharellales | Ceratobasidium | 358 | 473.7 | 0 | 91.99 |
| OTU_4568 | 17 | Sebacinales | - | 328 | 530.3 | 0 | 99.05 |
| OTU_4582* | 553 | Sebacinales | - | 352 | 423.9 | 0 | 89.64 |
| OTU_4657 | 5 | Sebacinales | - | 340 | 520 | 0 | 96.19 |
| OTU_4825* | 11 | Cantharellales | - | 412 | 145.8 | 0 | 73.48 |
| OTU_4850 | 5 | Sebacinales | - | 373 | 458.2 | 0 | 90.19 |
| OTU_4899 | 5 | Sebacinales | Serendipitaceae | 317 | 509.7 | 0 | 97.79 |
