# Supplementary material for: In situ Orchid Seedling-Trap Experiment Shows Few Keystone and Many Randomly Associated Mycorrhizal Fungal Species During Early Plant Colonization
Source: Front Plant Sci. 2018 Nov 16;9:1664. doi: 10.3389/fpls.2018.01664 (PMC6250785; doi:10.3389/fpls.2018.01664)
Supplement: Supplementary file 3 [file Table_3.DOCX]

Supplementary Material

*In Situ* Orchid Seedling-Trap Experiment Shows Few Keystone and Many Randomly-Associated Mycorrhizal Fungal Species During Early Plant Colonization

Stefania Cevallos, Stéphane Declerck, Juan Pablo Suárez *

*** Correspondence:** Juan Pablo Suárez: jpsuarez@utpl.edu.ec

**Supplementary Table 3** Similarity indices of mycorrhizal communities, estimated pairwise between the altitudinal levels of each treatment (T2: Transect T2, Q5: Transect Q5; C: *Cyrtochilum retusum,* E: *Epidendrum macrum*).

|  |  | T2C | | | | | T2E | | | | | Q5C | | | | |
| --- | --- | --- | --- | --- | --- | --- | --- | --- | --- | --- | --- | --- | --- | --- | --- | --- |
| 1st sample (1s) | 2nd sample (2s) | 1s OTUs | 2s OTUs | Shared | Chao-Jaccard | Chao-Sorensen | 1s OTUs | 2s OTUs | Shared | Chao-Jaccard | Chao-Sorensen | 1s OTUs | 2s OTUs | Shared | Chao-Jaccard | Chao-Sorensen |
| 1 | **2** | 6 | 7 | 3 | 0.214 | 0.352 | 2 | 2 | 0 | 0 | 0 | 5 | 4 | 0 | 0 | 0 |
| 1 | **3** | - | - | - | - | - | 2 | 3 | 1 | 0.839 | **0.912** | 5 | 21 | 5 | 1 | 1 |
| 1 | **4** | 6 | 7 | 2 | 0.41 | **0.582** | 2 | 2 | 0 | 0 | 0 | 5 | 5 | 0 | 0 | 0 |
| 1 | **5** | 6 | 10 | 0 | 0 | 0 | 2 | 3 | 0 | 0 | 0 | 5 | 5 | 1 | 0 | 0 |
| 1 | **6** | 6 | 18 | 2 | 0.27 | 0.425 | 2 | 4 | 0 | 0 | 0 | 5 | 2 | 0 | 0 | 0 |
| 1 | **7** | 6 | 8 | 2 | 0.664 | 0.798 | - | - | - | - | - | 5 | 8 | 2 | 0.049 | 0.093 |
| 1 | **8** | 6 | 10 | 0 | 0 | 0 | 2 | 7 | 0 | 0 | 0 | 5 | 5 | 4 | 0.917 | **0.957** |
| 1 | **9** | 6 | 11 | 2 | 0.177 | 0.301 | 2 | 13 | 0* | 0 | 0 | 5 | 5 | 3 | 0.278 | 0.435 |
| 1 | **10** | 6 | 2 | 0 | 0 | 0 | 2 | 3 | 0 | 0 | 0 | - | - | - | - | - |
| 2 | **3** | - | - | - | - | - | 2 | 3 | 0 | 0 | 0 | 4 | 21 | 1 | 0.002 | 0.003 |
| 2 | **4** | 7 | 7 | 4 | 0.687 | **0.815** | 2 | 2 | 0 | 0 | 0 | 4 | 5 | 0 | 0 | 0 |
| 2 | **5** | 7 | 10 | 2 | 0.469 | **0.639** | 2 | 3 | 2 | 0.057 | 0.107 | 4 | 5 | 1 | 0.001 | 0.002 |
| 2 | **6** | 7 | 18 | 4 | 0.438 | **0.609** | 2 | 4 | 0 | 0 | 0 | 4 | 2 | 1 | 0.013 | 0.026 |
| 2 | **7** | 7 | 8 | 2 | 0.192 | 0.322 | - | - | - | - | - | 4 | 8 | 0 | 0 | 0 |
| 2 | **8** | 7 | 10 | 1 | 0.034 | 0.066 | 2 | 7 | 2 | 0.055 | 0.104 | 4 | 5 | 0 | 0 | 0 |
| 2 | **9** | 7 | 11 | 4 | 0.353 | **0.522** | 2 | 13 | 1 | 0 | 0 | 4 | 5 | 0 | 0 | 0 |
| 2 | **10** | 7 | 2 | 0 | 0 | 0 | 2 | 3 | 2 | 0.351 | 0.52 | - | - | - | - | - |
| 3 | **4** | - | - | - | - | - | 3 | 2 | 1 | 0.01 | 0.02 | 21 | 5 | 1 | 0 | 0.001 |
| 3 | **5** | - | - | - | - | - | 3 | 3 | 0 | 0 | 0 | 21 | 5 | 3 | 0.001 | 0.002 |
| 3 | **6** | - | - | - | - | - | 3 | 4 | 0 | 0 | 0 | 21 | 2 | 2 | 0.002 | 0.005 |
| 3 | **7** | - | - | - | - | - | - | - | - | - | - | 21 | 8 | 4 | 0.062 | 0.117 |
| 3 | **8** | - | - | - | - | - | 3 | 7 | 1 | 0.002 | 0.003 | 21 | 5 | 5 | 0.901 | **0.948** |
| 3 | **9** | - | - | - | - | - | 3 | 13 | 0 | 0 | 0 | 21 | 5 | 4 | 0.45 | 0.621 |
| 3 | **10** | - | - | - | - | - | 3 | 3 | 0 | 0 | 0 | - | - | - | - | - |
| 4 | **5** | 7 | 10 | 3 | 0.277 | 0.434 | 2 | 3 | 0 | 0 | 0 | 5 | 5 | 0 | 0 | 0 |
| 4 | **6** | 7 | 18 | 5 | 0.465 | **0.635** | 2 | 4 | 0 | 0 | 0 | 5 | 2 | 1 | 0 | 0.001 |
| 4 | **7** | 7 | 8 | 2 | 0.34 | **0.507** | - | - | - | - | - | 5 | 8 | 1 | 0.001 | 0.001 |
| 4 | **8** | 7 | 10 | 0 | 0 | 0 | 2 | 7 | 1 | 0.002 | 0.004 | 5 | 5 | 0 | 0 | 0 |
| 4 | **9** | 7 | 11 | 4 | 0.325 | 0.491 | 2 | 13 | 0 | 0 | 0 | 5 | 5 | 0 | 0 | 0 |
| 4 | **10** | 7 | 2 | 0 | 0 | 0 | 2 | 3 | 0 | 0 | 0 | - | - | - | - | - |
| 5 | **6** | 10 | 18 | 7 | 0.399 | **0.57** | 3 | 4 | 0 | 0 | 0 | 5 | 2 | 1 | 0.001 | 0.002 |
| 5 | **7** | 10 | 8 | 2 | 0.012 | 0.024 | - | - | - | - | - | 5 | 8 | 1 | 0 | 0 |
| 5 | **8** | 10 | 10 | 3 | 0.022 | 0.043 | 3 | 7 | 2 | 0.029 | 0.056 | 5 | 5 | 1 | 0 | 0 |
| 5 | **9** | 10 | 11 | 4 | 0.186 | 0.314 | 3 | 13 |  | 0 | 0 | 5 | 5 | 1 | 0 | 0 |
| 5 | **10** | 10 | 2 | 1 | 0.002 | 0.004 | 3 | 3 | 2 | 0.055 | 0.104 | - | - | - | - | - |
| 6 | **7** | 18 | 8 | 4 | 0.435 | 0.606 | - | - | - | - | - | 2 | 8 | 1 | 0.005 | 0.01 |
| 6 | **8** | 18 | 10 | 2 | 0.047 | 0.09 | 4 | 7 | 2 | 0.013 | 0.025 | 2 | 5 | 0 | 0 | 0 |
| 6 | **9** | 18 | 11 | 5 | 0.299 | 0.46 | 4 | 13 | 3 | 0.984 | **0.992** | 2 | 5 | 0 | 0 | 0 |
| 6 | **10** | 18 | 2 | 1 | 0.002 | 0.005 | 4 | 3 | 0 | 0 | 0 | - | - | - | - | - |
| 7 | **8** | 8 | 10 | 4 | 0.183 | 0.309 | - | - | - | - | - | 8 | 5 | 2 | 0.038 | 0.074 |
| 7 | **9** | 8 | 11 | 4 | 0.183 | 0.31 | - | - | - | - | - | 8 | 5 | 1 | 0.007 | 0.014 |
| 7 | **10** | 8 | 2 | 1 | 0.002 | 0.005 | - | - | - | - | - | - | - | - | - | - |
| 8 | **9** | 10 | 11 | 4 | 0.02 | 0.04 | 7 | 13 | 4 | 1 | 1 | 5 | 5 | 3 | 0.277 | 0.434 |
| 8 | **10** | 10 | 2 | 1 | 0.002 | 0.005 | 7 | 3 | 2 | 0.055 | 0.104 | - | - | - | - | - |
| 9 | **10** | 11 | 2 | 1 | 0.002 | 0.004 | 13 | 3 | 1 | 0 | 0 | - | - | - | - | - |
