# Supplementary material for: In situ Orchid Seedling-Trap Experiment Shows Few Keystone and Many Randomly Associated Mycorrhizal Fungal Species During Early Plant Colonization
Source: Front Plant Sci. 2018 Nov 16;9:1664. doi: 10.3389/fpls.2018.01664 (PMC6250785; doi:10.3389/fpls.2018.01664)
Supplement: Supplementary file 4 [file Table_4.DOCX]

Supplementary Material

*In Situ* Orchid Seedling-Trap Experiment Shows Few Keystone and Many Randomly-Associated Mycorrhizal Fungal Species During Early Plant Colonization

Stefania Cevallos, Stéphane Declerck, Juan Pablo Suárez *

*** Correspondence:** Juan Pablo Suárez: jpsuarez@utpl.edu.ec

**Supplementary Table 4** Number of operational taxonomic units (OTUs) identified in T2CS1 and T2CS2 treatments (T2: Transect 2; C: *Cyrtochilum macrum*; S1: 1^st^ sampling, S2: 2^nd^ sampling) and similarity indices of mycorrhizal fungal communities identified in function of the temporal variation.

|  | T2CS1 | T2CS2 | Shared OTUs | Chao-Sorence | Chao-Jaccard | P value |
| --- | --- | --- | --- | --- | --- | --- |
| A1 | 6 | 14 | 2 | 0.111 | 0.2 | 0.006 |
| A2 | 7 | 5 | 2 | 0.1 | 0.182 | 0.346 |
| A3 | - | - | - | - | - | - |
| A4 | 7 | 11 | 1 | 0.063 | 0.118 | 0.180 |
| A5 | 10 | 22 | 5 | 0.077 | 0.143 | 0.001 |
| A6 | 18 | 19 | 6 | 0.042 | 0.08 | 0.753 |
| A7 | 8 | 19 | 4 | 0 | 0 | 0.001 |
| A8 | 10 | - | - | - | - | - |
| A9 | 11 | 25 | 7 | 0.069 | 0.129 | 0 |
| A10 | 2 | 19 | 1 | 0.087 | 0.16 | 0 |
