# Supplementary material for: In situ Orchid Seedling-Trap Experiment Shows Few Keystone and Many Randomly Associated Mycorrhizal Fungal Species During Early Plant Colonization
Source: Front Plant Sci. 2018 Nov 16;9:1664. doi: 10.3389/fpls.2018.01664 (PMC6250785; doi:10.3389/fpls.2018.01664)
Supplement: Supplementary file 5 [file Data_Sheet_1.docx]

Supplementary Material

*In Situ* Orchid Seedling-Trap Experiment Shows Few Keystone and Many Randomly-Associated Mycorrhizal Fungal Species During Early Plant Colonization

Stefania Cevallos, Stéphane Declerck, Juan Pablo Suárez *

*** Correspondence:** Juan Pablo Suárez: jpsuarez@utpl.edu.ec

**Supplementary data** Analysis outputs of samples rarefied to the 10% of the highest sequence reads per sample. We used seqtk software in order to process the random sequences subsampling.

The non-rarefied outputs are available in the main document of this manuscript.

**Fig. 1** Frequency distribution of the identified fungal orders of the orchid mycorrhizal fungi identified in association with *Cyrtochilum retusum* and *Epidendrum macrum* after rarefaction to the 10% of the highest sequence reads per sample. Note that orders of orchid mycorrhizal fungi had similar frequency distribution as no-rarefied data. In both, rarefied and no-rarefied analysis the dominant order was Cantharellales.

**Fig. 2** Rarefaction curves of orchid mycorrhizal fungi OTU (operational taxonomic unit) richness in four treatments of the seedling-trap experiment after rarefaction to the 10% of the highest sequence reads per sample. T2: site T2; Q5: site 5; C: *Cyrtochilum retusum*; E: *Epidendrum macrum*; S1: 1^st^ sampling and S2: 2^nd^ sampling. Note the same patterns of OTU richness between rarefied and no-rarefied analysis.

**Fig. 3** Non-multidimensional scaling (NMDS) plot of orchid mycorrhizal fungal communities associated with *Cyrtochilum retusum* at T2 (black dots) and Q5 (white squares) sites, after rarefaction to the 10% of the highest sequence reads per sample (stress value = 0. 070516, p-value 0.2645). Same results as in no-rarefied data.


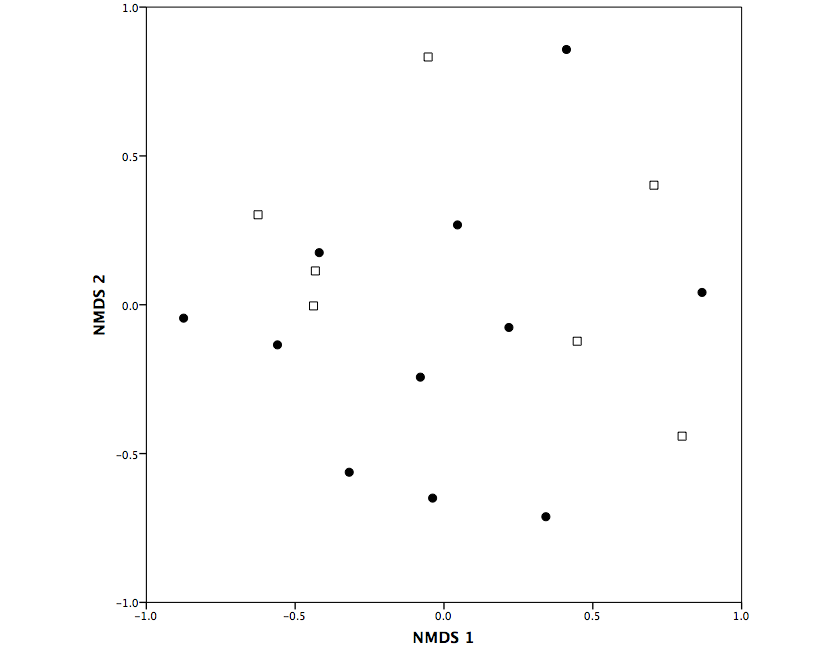


**Fig. 4** Non-multidimensional scaling (NMDS) plot of mycorrhizal fungi detected in *Cyrtochilum macrum* sampled at two colonization times, after rarefaction to the 10% of the highest sequence reads per sample (stress value = 0. 05375). The black dots correspond to samples obtained from the first sampling (T2CS1 treatment) and the white triangles correspond to the second sampling (T2CS2 treatment), after three months and one year, respectively, of the assay was established. Note that OMF communities shown low similarity as in no-rarefied data.


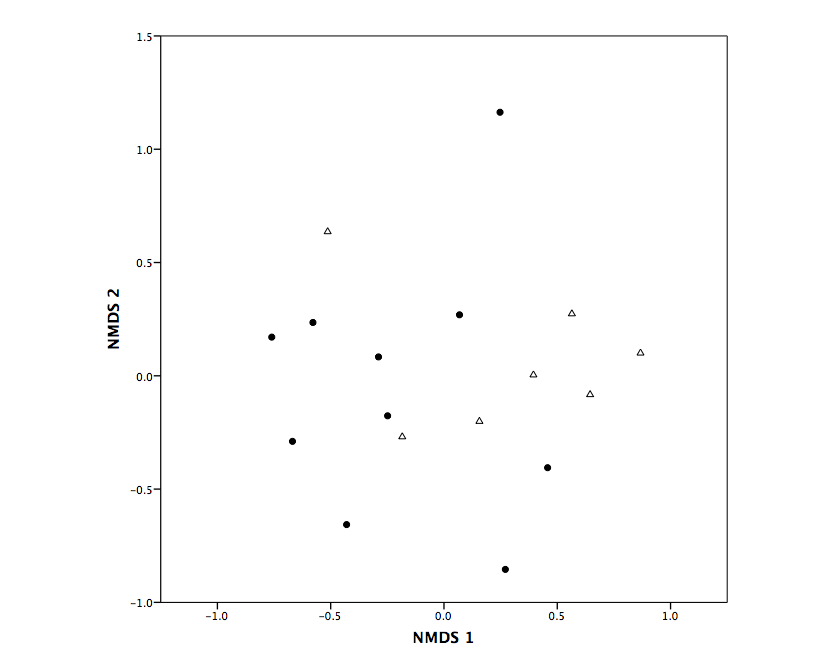


**Table 1** Number of orchid mycorrhizal fungi (OMF) OTUs and the similarity indices for the co-occurring orchids *Cyrtochilum retusum* and *Epidendrum macrum* at the different elevational levels (A1-A10)*,* after rarefaction to the 10% of the highest sequence reads per sample. Note that *Cyrtochilum retusum* and *Epidendrum macrum* showed low similarity at both analysis outputs. The *p-value* (0.0862) supported the significant difference between the co-existing orchid species in both rarefied and non-rarefied analysis.

|  | OMF-OTUs  from  Cyrtochilum retusum | OMF-OTUs  from  Epidendrum macrum | Shared  OMF-OTUs | Chao-Jaccard | Chao-Sorensen |  |
| --- | --- | --- | --- | --- | --- | --- |
| **A1** | 2 | 0 | 0 | 0 | 0 |  |
| **A2** | 4 | 1 | 1 | 0.25 | 0.4 |  |
| **A4** | 4 | 1 | 0 | 0 | 0 |  |
| **A5** | 9 | 2 | 1 | 0.1 | 0.182 |  |
| **A6** | 4 | 3 | 2 | 0.4 | 0.571 |  |
| **A8** | 6 | 3 | 1 | 0.125 | 0.222 |  |
| **A9** | 1 | 5 | 0 | 0 | 0 |  |
| **A10** | 0 | 2 | 0 | 0 | 0 |  |

**Table 2** Collected seedling-traps and number of identified mycorrhizal and non-mycorrhizal OTUs, after rarefaction to the 10% of the highest sequence reads per sample. T2: Transect T2, Q5: Transect Q5; C: *Cyrtochilum retusum,* E: *Epidendrum macrum*; S1: 1^st^ sampling, S2: 2^nd^ sampling and A1-A10: elevational levels. Note that the number of OTUs per seedling-trap decrease after the samples rarefaction.

| **Sampled seedling-trap** | **Mycorrhizal OTUs** |  | **Non-mycorrhizal OTUs** |
| --- | --- | --- | --- |
| T2CS1_A1 | 2 |  | 52 |
| T2CS1_A2 | 4 |  | 67 |
| T2CS1_A3 | - |  | - |
| T2CS1_A4 | 5 |  | 91 |
| T2CS1_A5 | 4 |  | 95 |
| T2CS1_A6 | 9 |  | 105 |
| T2CS1_A7 | 4 |  | 121 |
| T2CS1_A8 | 5 |  | 113 |
| T2CS1_A9 | 6 |  | 102 |
| T2CS1_A10 | 1 |  | 57 |
| T2ES1_ A1 | 0 |  | 77 |
| T2ES1_ A2 | 1 |  | 71 |
| T2ES1_ A3 | 1 |  | 66 |
| T2ES1_ A4 | 1 |  | 117 |
| T2ES1_ A5 | 2 |  | 76 |
| T2ES1_ A6 | 3 |  | 61 |
| T2ES1_A7 | - |  | - |
| T2ES1_A8 | 3 |  | 99 |
| T2ES1_ A9 | 5 |  | 64 |
| T2ES1_ A10 | 2 |  | 139 |
| Q5CS1_ A1 | - |  | - |
| Q5CS1_ A2 | 2 |  | 139 |
| Q5CS1_ A3 | 1 |  | 64 |
| Q5CS1_ A4 | 6 |  | 99 |
| Q5CS1_ A5 | 1 |  | 77 |
| Q5CS1_ A6 | 3 |  | 71 |
| Q5CS1_ A7 | 2 |  | 66 |
| Q5CS1_ A8 | 2 |  | 117 |
| Q5CS1_ A9 | 3 |  | 76 |
| Q5CS1_ A10 | 4 |  | 61 |
| T2CS2_ A1 | 6 |  | 82 |
| T2CS2_ A2 | 4 |  | 77 |
| T2CS2_ A3 | - |  | - |
| T2CS2_ A4 | 3 |  | 115 |
| T2CS2_ A5 | 5 |  | 72 |
| T2CS2_ A6 | 9 |  | 131 |
| T2CS2_ A7 | 7 |  | 137 |
| T2CS2_ A8 | - |  | - |
| T2CS2_ A9 | 12 |  | 224 |
| T2CS2_ A10 | 7 |  | 140 |

**Table 3** Operational taxonomic units putatively assigned to mycorrhizal fungi using UNITE database, after rarefaction to the 10% of the highest sequence reads per sample. * sequences identity lower than 90%.

| OTU Id | Number of sequences | Order | Family/Genus | Length (bp) | Score | E-value | Sequence identity % |
| --- | --- | --- | --- | --- | --- | --- | --- |
| OTU_28 | 2660 | Cantharellales | Ceratobasidiaceae | 360 | 568.1 | 0 | 99.12 |
| OTU_43 | 840 | Cantharellales | - | 362 | 523.5 | 0 | 94.25 |
| OTU_47* | 347 | Sebacinales | - | 329 | 413.6 | 0 | 89.86 |
| OTU_50 | 759 | Sebacinales | Serendipitaceae | 317 | 509.7 | 0 | 97.79 |
| OTU_64 | 681 | Cantharellales | Ceratobasidium | 446 | 652.2 | 0 | 94.87 |
| OTU_80 | 241 | Sebacinales | - | 348 | 468.5 | 0 | 92.57 |
| OTU_179* | 193 | Cantharellales | - | 382 | 152.6 | 0 | 74.41 |
| OTU_193 | 55 | Serendipita | Serendipita | 459 | 130.3 | 0 | 94.44 |
| OTU_231 | 143 | Sebacinales | - | 336 | 477.1 | 0 | 94.07 |
| OTU_241 | 46 | Sebacinales | - | 341 | 525.2 | 0 | 96.48 |
| OTU_244 | 37 | Cantharellales | - | 347 | 94.3 | 0 | 92.75 |
| OTU_251 | 484 | Cantharellales | Ceratobasidiaceae | 353 | 542.4 | 0 | 99.38 |
| OTU_259 | 28 | Sebacinales | - | 335 | 566.4 | 0 | 99.4 |
| OTU_261 | 103 | Cantharellales | Ceratobasidium | 374 | 545.8 | 0 | 94.92 |
| OTU_318 | 109 | Cantharellales | - | 410 | 595.6 | 0 | 94.87 |
| OTU_352 | 13 | Cantharellales | Ceratobasidiaceae | 358 | 502.9 | 0 | 93.85 |
| OTU_357 | 45 | Sebacinales | - | 412 | 648.8 | 0 | 97.09 |
| OTU_499 | 8 | Cantharellales | - | 331 | 80.5 | 0 | 91.8 |
| OTU_530 | 8 | Sebacinales | - | 334 | 554.4 | 0 | 98.8 |
| OTU_549 | 10 | Cantharellales | Ceratobasidium | 363 | 559.5 | 0 | 96.43 |
| OTU_552 | 10 | Atractiellales | - | 354 | 609.3 | 0 | 100 |
| OTU_574* | 11 | Cantharellales | - | 383 | 116.6 | 0 | 72.51 |
| OTU_582 | 6 | Sebacinales | - | 340 | 544.1 | 0 | 97.65 |
| OTU_599* | 10 | Cantharellales | - | 383 | 84 | 0 | 70.83 |
| OTU_611 | 14 | Cantharellales | - | 433 | 690 | 0 | 97.68 |
| OTU_632 | 8 | Sebacinales | - | 334 | 569.8 | 0 | 99.7 |
| OTU_675* | 6 | Cantharellales | - | 433 | 401.6 | 0 | 87.6 |
| OTU_738 | 6 | Cantharellales | Ceratobasidiaceae | 357 | 542.4 | 0 | 96.08 |
| OTU_749 | 24 | Cantharellales | Ceratobasidium | 418 | 662.5 | 0 | 97.37 |
| OTU_787 | 8 | Cantharellales | Ceratobasidiaceae | 441 | 702 | 0 | 97.51 |
| OTU_854 | 13 | Sebacinales | Sebacinaceae | 339 | 521.7 | 0 | 96.46 |
| OTU_870 | 2 | Atractiellales | - | 346 | 585.3 | 0 | 99.42 |
| OTU_879* | 8 | Cantharellales | - | 382 | 102.8 | 0 | 71.88 |
| OTU_913 | 10 | Cantharellales | - | 442 | 571.5 | 0 | 96.26 |
| OTU_973 | 9 | Cantharellales | - | 361 | 116.6 | 0 | 91.21 |
| OTU_980* | 17 | Cantharellales | - | 383 | 144.1 | 0 | 74.01 |
| OTU_1067 | 27 | Cantharellales | - | 347 | 94.3 | 0 | 92.75 |
| OTU_1087 | 5 | Sebacinales | - | 329 | 425.6 | 0 | 91.32 |
| OTU_1197* | 8 | Sebacinales | Sebacinaceae | 280 | 473.7 | 0 | 87.94 |
| OTU_1203 | 5 | Cantharellales | - | 335 | 550.9 | 0 | 98.51 |
| OTU_1277 | 3 | Sebacinales | - | 366 | 609.3 | 0 | 98.91 |

**Table 4** Similarity indices of mycorrhizal communities, estimated pairwise between the altitudinal levels of each treatment (T2: Transect T2, Q5: Transect Q5; C: *Cyrtochilum retusum,* E: *Epidendrum macrum*) after rarefaction to the 10% of the highest sequence reads per sample. Note that no clear patterns were observed along elevational levels in both rarefied and no-rarefy analysis.

|  |  | T2C | | | | | T2E | | | | | Q5C | | | | |
| --- | --- | --- | --- | --- | --- | --- | --- | --- | --- | --- | --- | --- | --- | --- | --- | --- |
| 1st sample (1s) | 2nd sample (2s) | 1s OTUs | 2s OTUs | Shared | Chao-Jaccard | Chao-Sorensen | 1s OTUs | 2s OTUs | Shared | Chao-Jaccard | Chao-Sorensen | 1s OTUs | 2s OTUs | Shared | Chao-Jaccard | Chao-Sorensen |
| 1 | **2** | 2 | 4 | 1 | 0.2 | 0.333 | 0 | 1 | 0 | 0 | 0 | 1 | 3 | 0 | 0 | 0 |
| 1 | **3** | - | - | - | - | - | 0 | 1 | 0 | 0 | 0 | 1 | 2 | 0 | 0 | 0 |
| 1 | **4** | 2 | 5 | 2 | 0.4 | 0.571 | 0 | 1 | 0 | 0 | 0 | 1 | 2 | 0 | 0 | 0 |
| 1 | **5** | 2 | 4 | 0 | 0 | 0 | 0 | 2 | 0 | 0 | 0 | 1 | 3 | 0 | 0 | 0 |
| 1 | **6** | 2 | 9 | 2 | 0.222 | 0.364 | 0 | 3 | 0 | 0 | 0 | 1 | 4 | 0 | 0 | 0 |
| 1 | **7** | 2 | 4 | 2 | 0.5 | 0.667 | - | - | - | - | - | 1 | 6 | 0 | 0 | 0 |
| 1 | **8** | 2 | 5 | 0 | 0 | 0 | 0 | 3 | 0 | 0 | 0 | 1 | 1 | 0 | 0 | 0 |
| 1 | **9** | 2 | 6 | 2 | 0.333 | 0.5 | 0 | 5 | 0 | 0 | 0 | 1 | 2 | 0 | 0 | 0 |
| 1 | **10** | 2 | 1 | 0 | 0 | 0 | 0 | 2 | 0 | 0 | 0 | - | - | - | - | - |
| 2 | **3** | - | - | - | - | - | 1 | 1 | 0 | 0 | 0 | 3 | 2 | 1 | 0.345 | 0.4 |
| 2 | **4** | 4 | 5 | 2 | 0.286 | 0.444 | 1 | 1 | 0 | 0 | 0 | 3 | 2 | 0 | 0 | 0 |
| 2 | **5** | 4 | 4 | 1 | 0.143 | 0.25 | 1 | 2 | 1 | 0.5 | 0.667 | 3 | 3 | 1 | 0.286 | 0.333 |
| 2 | **6** | 4 | 9 | 2 | 0.182 | 0.308 | 1 | 3 | 0 | 0 | 0 | 3 | 4 | 0 | 0 | 0 |
| 2 | **7** | 4 | 4 | 1 | 0.143 | 0.25 | - | - | - | - | - | 3 | 6 | 1 | 0.178 | 0.222 |
| 2 | **8** | 4 | 5 | 1 | 0.125 | 0.222 | 1 | 3 | 1 | 0.333 | 0.5 | 3 | 1 | 0 | 0 | 0 |
| 2 | **9** | 4 | 6 | 2 | 0.25 | 0.4 | 1 | 5 | 0 | 0 | 0 | 3 | 2 | 1 | 0.345 | 0.4 |
| 2 | **10** | 4 | 1 | 0 | 0 | 0 | 1 | 2 | 1 | 0.5 | 0.667 | - | - | - | - | - |
| 3 | **4** | - | - | - | - | - | 1 | 1 | 0 | 0 | 0 | 2 | 2 | 0 | 0 | 0 |
| 3 | **5** | - | - | - | - | - | 1 | 2 | 0 | 0 | 0 | 2 | 3 | 0 | 0 | 0 |
| 3 | **6** | - | - | - | - | - | 1 | 3 | 0 | 0 | 0 | 2 | 4 | 0 | 0 | 0 |
| 3 | **7** | - | - | - | - | - | - | - | - | - | - | 2 | 6 | 0 | 0 | 0 |
| 3 | **8** | - | - | - | - | - | 1 | 3 | 0 | 0 | 0 | 2 | 1 | 0 | 0 | 0 |
| 3 | **9** | - | - | - | - | - | 1 | 5 | 0 | 0 | 0 | 2 | 2 | 0 | 0 | 0 |
| 3 | **10** | - | - | - | - | - | 1 | 2 | 0 | 0 | 0 | - | - | - | - | - |
| 4 | **5** | 5 |  | 1 | 0.125 | 0.222 | 1 | 2 | 0 | 0 | 0 | 2 | 3 | 0 | 0 | 0 |
| 4 | **6** | 5 | 9 | 4 | 0.4 | 0.571 | 1 | 3 | 0 | 0 | 0 | 2 | 4 | 0 | 0 | 0 |
| 4 | **7** | 5 | 4 | 2 | 0.286 | 0.444 | - | - | - | - | - | 2 | 6 | 0 | 0 | 0 |
| 4 | **8** | 5 | 5 | 0 | 0 | 0 | 1 | 3 | 0 | 0 | 0 | 2 | 1 | 0 | 0 | 0 |
| 4 | **9** | 5 | 6 | 3 | 0.375 | 0.545 | 1 | 5 | 0 | 0 | 0 | 2 | 2 | 0 | 0 | 0 |
| 4 | **10** | 5 | 1 | 0 | 0 | 0 | 1 | 2 | 0 | 0 | 0 | - | - | - | - | - |
| 5 | **6** | 4 | 9 | 3 | 0.3 | 0.462 | 2 | 3 | 0 | 0 | 0 | 3 | 4 | 2 | 0.833 | 0.571 |
| 5 | **7** | 4 | 4 | 1 | 0.143 | 0.25 | - | - | - | - | - | 3 | 6 | 2 | 0.556 | 0.444 |
| 5 | **8** | 4 | 5 | 1 | 0.125 | 0.222 | 2 | 3 | 1 | 0.25 | 0.4 | 3 | 1 | 0 | 0 | 0 |
| 5 | **9** | 4 | 6 | 2 | 0.25 | 0.4 | 2 | 5 | 0 | 0 | 0 | 3 | 2 | 2 | 1 | 0.8 |
| 5 | **10** | 4 | 1 | 0 | 0 | 0 | 2 | 2 | 1 | 0.333 | 0.5 | - | - | - | - | - |
| 6 | **7** | 9 | 4 | 3 | 0.3 | 0.462 | - | - | - | - | - | 4 | 6 | 2 | 0.554 | 0.4 |
| 6 | **8** | 9 | 5 | 1 | 0.077 | 0.143 | 3 | 3 | 0 | 0 | 0 | 4 | 1 | 0 | 0 | 0 |
| 6 | **9** | 9 | 6 | 4 | 0.364 | 0.533 | 3 | 5 | 2 | 0.333 | 0.5 | 4 | 2 | 1 | 0.274 | 0.333 |
| 6 | **10** | 9 | 1 | 0 | 0 | 0 | 3 | 2 | 0 | 0 | 0 | - | - | - | - | - |
| 7 | **8** | 4 | 5 | 2 | 0.286 | 0.444 | - | - | - | - | - | 6 | 1 | 0 | 0 | 0 |
| 7 | **9** | 4 | 6 | 3 | 0.429 | 0.6 | - | - | - | - | - | 6 | 2 | 2 | 0.5 | 0.5 |
| 7 | **10** | 4 | 1 | 0 | 0 | 0 | - | - | - | - | - | - | - | - | - | - |
| 8 | **9** | 5 | 6 | 1 | 0.1 | 0.182 | 3 | 5 | 0 | 0 | 0 | 1 | 2 | 0 | 0 | 0 |
| 8 | **10** | 5 | 1 | 0 | 0 | 0 | 3 | 2 | 1 | 0.25 | 0.4 | - | - | - | - | - |
| 9 | **10** | 6 | 1 | 0 | 0 | 0 | 5 | 2 | 0 | 0 | 0 | - | - | - | - | - |

**Table 5** Number of operational taxonomic units (OTUs) identified in T2CS1 and T2CS2 treatments (T2: Transect 2; C: *Cyrtochilum macrum*; S1: 1^st^ sampling, S2: 2^nd^ sampling) and similarity indices of mycorrhizal fungal communities identified in function of the temporal variation, after rarefaction to the 10% of the highest sequence reads per sample. Same results as in no-rarefied data, the richness of the orchid mycorrhizal fungi (OMF) communities increased after one year of the seedling-trap establishment and low similarity between OMF communities was observed.

|  | T2CS1 | T2CS2 | Shared OTUs | Chao-Sorence | Chao-Jaccard | P value |
| --- | --- | --- | --- | --- | --- | --- |
| A1 | 2 | 6 | 1 | 0.143 | 0.250 | 0.140 |
| A2 | 4 | 4 | 2 | 0.333 | 0.500 | 1.000 |
| A3 | - | - | - | - | - | - |
| A4 | 5 | 3 | 0 | 0 | 0 | 0.463 |
| A5 | 4 | 5 | 2 | 0.286 | 0.444 | 0.728 |
| A6 | 9 | 9 | 5 | 0.385 | 0.556 | 1 |
| A7 | 4 | 7 | 1 | 0.100 | 0.182 | 0.337 |
| A8 | 5 | - | - | - | - | - |
| A9 | 6 | 12 | 4 | 0.286 | 0.444 | 0.112 |
| A10 | 1 | 7 | 0 | 0 | 0 | 0.26 |
